# Apoptosis and apoptotic pathway in actinic prurigo by immunohistochemistry

**DOI:** 10.4317/medoral.20765

**Published:** 2015-11-30

**Authors:** Juan-Carlos Cuevas-González, María-Elisa Vega-Memíje, Francisco-Javier García-Vázquez, Erika Rodríguez-Lobato, José-Eduardo Farfán-Morales

**Affiliations:** 1 Oral Pathology Laboratory, National Autonomous University of Mexico, Mexico City, Mexico; 2 Department of Dermatology, Dr. Manuel Gea González General Hospital, Mexico City, Mexico; 3 Molecular Pathology Laboratory, National Institute of Pediatrics, Mexico City, Mexico; 4 Southern associated Pathologists “Specialists in pathology laboratories” Mexico City, Mexico

## Abstract

**Background:**

Actinic prurigo (AP) is an idiopathic photodermatosis, this entity requires exposure to UV-B and -A to develop lesions. Apoptosis is a physiological death program that can be initiated by a permanently active mechanism (extrinsic pathway) or irreparable damage (intrinsic pathway).

**Material and Methods:**

Descriptive study, the sample size comprised 64 paraffin blocks of tissue with a diagnosis of AP. In H&E-stained slides, the diagnosis of AP was corroborated, and 1-µm-thick sections were processed for immunohistochemistry (IHC). A database was constructed with SPSS version 20, Inc., Chicago, IL, USA, and descriptive statistics were analyzed by X2 test and comparison of means.

**Results:**

A total of 64 cases were processed, of which 40 (62.5%) were cheilitis AP and 24 (37.5%) were AP in the skin. Of the 40 cheilitis samples, 27 were positive for Bcl-2 and caspase 3 (67.5%), p53 was expressed in 30 (75%). Of the skin lesions,p53 and caspase 3 were expressed in 18 of 24 cases (75%), and 13 were positive for Bcl-2 (54%).

**Conclusions:**

We propose that apoptosis is the last step in the type IV subtype a-b hypersensitivity response-activation of the intrinsic pathway indicates that external factors, such as UV-A and -B are the trigger.

**Key words:**Apoptosis, actinic prurigo, cheilitis actinic prurigo.

## Introduction

Actinic prurigo (AP) is an idiopathic photodermatosis, the first manifestations of which occur during childhood, and predominantly affects women. With regard to genetic susceptibility, a strong link has been reported between AP and human leukocyte antigen (HLA), particularly with the HLA-DR4, (1) allele, which varies between populations. In Mexico, 90% to 92.8% of patients with AP have this allele (2-4). HLA-DRB1*0407 (5,6) is the most common subtype (60% to 80%) (2-4).

AP is characterized by symmetrical and bilateral lesions in sun-exposed areas, such as the face, neck, trunk, upper and lower extremities, lips, and conjunctiva. As a photodermatosis, this entity requires exposure to UV-B and -A to develop lesions and presents clinically as macules, papules, excoriations, serohematic crusts, areas of lichenification, scarring, and residual hypo- or hyper pigmentation (2,6-8). Based on their histological characteristics, the skin lesions can be considered hyperkeratosis; parakeratosis; acanthosis; thickening of the basal lamina; perivascular inflammatory infiltrate; and nodular lymphocyte formations, eosinophils, and mast cells. In cheilitis, hyperkeratosis, acanthosis, spongiosis and vacuolization of the basal layer, eosinophils, melanophages, angiogenesis, edema, and formation of lymphoid follicles are observed, all of which pathognomonic of the disease (9).

The pathophysiology of AP has been examined with regard to immune and inflammatory responses and has been proposed as a type IV, subtype b hypersensitivity reaction; the inflammatory infiltrate comprises primarily mainly CD45RO, interleukin-2, and T cells (8). Moncada studied 16 patients with AP and found that T cell levels increased in peripheral blood versus controls, suggesting that an abnormal immune response causes the tissue damage in these patients (10).

In this condition, a reaction with type IV subtype “a” component entails a Th1 response in which macrophages are activated by secreting large amounts of interferon gamma and direct the production of complement-fixing antibodies.

In AP, TNF-α is expressed primarily mainly in keratinocytes in the suprabasal layer. UV-B light stimulates the production of TNF-α in keratinocytes, which rises to concentrations that can induce necrosis. In inflammatory diseases, TNF-α, in combination with interferon gamma, upregulates adhesion molecules on keratinocytes, as reported by researchers at our hospital, who measured syndecan-1 and E-cadherin expression in epithelial tissues with AP, indicating that although this disease has a strong inflammatory component, the expression of adhesion molecules is preserved. Another function of TNF-α is to stimulate fibroblast proliferation and capillary formation. The involvement of TNF-α has been demonstrated indirectly, based on the clinical improvement that occurs on inhibition by thalidomide (9).

In addition to a Th2 response, which develops in the type IV response, subtype “B” cells produce cytokines, such as IL3, IL4, IL5, and GM-CSF, of which IL4 is crucial for the activation of IgE (8).

We reported enhanced serum IgE levels in patients with AP who had moderate to severe injuries by (Access total IGE), compared with those with minor injuries, in whom the levels were within the normal range. Our working group identified tissue with AP that harbored mast cells and eosinophils by immunohistochemistry.

Thus, with regard to the components of the subtype “a” and “b” type IV responses, apoptosis occurs after the activation of cells and cytokines-a process that has not been demonstrated in the pathophysiology of AP.

Apoptosis is programmed cell death, in which a several enzymatic reactions are activated, leading to disorganization of protein networks and subsequent damage to DNA. In the initial phase of apoptosis, membrane integrity is maintained, preventing intracellular components from being released and tissue from incurring damage directly (9).

In this study the aim was evaluate the activation of apoptosis in AP and the apoptotic pathways that are involved.

## Material and Methods

This descriptive study was conducted in the Department of Dermatology, General Hospital Dr. Manuel Gea González and was approved by the ethics committee and research, patients signed an informed consent, the sample size comprised 64 paraffin blocks of tissue with a diagnosis of AP-40 corresponding to AP in the lip and 24 in the skin.

In H&E-stained slides, the diagnosis of AP was corroborated by 2 examiners, and 1- µm-thick sections were processed for immunohistochemistry (IHC). The slides were deparaffinized and rehydrated, antigen retrieval was performed with 0.1% sodium citrate, pH 6.2, endogenous peroxidase was inactivated with 0.9% hydrogen peroxide, and the slides were washed in distilled water and allowed to stand for 5 minutes in phosphate-buffered saline (PBS).

The tissue sections were incubated for 45 minutes with the following antibodies: bcl-2 (1:100, Dako clone 124), p53 (1:100, Dako clone DO-7), caspase 3 (1:100, Biocare), Bax (1:100, Dako), and Fas-CD-95 (1:100, Santa Cruz clone C-20). Then, secondary anti-mouse or -rabbit and streptavidin/peroxidase were added sequentially for 30 minutes each. The reaction was visualized with diaminobenzidine (Dako) and stained with Hill’s hematoxylin.

The immunoreactivity as evaluated in 2 phases: first by 2 evaluators to determine the positive histological sites and second to measure the intensity of the reactions semi quantitatively using Image Lab, version 2.0. Four photos (400x) were taken for each positive case, for which the optical density was measured and expressed as the mean for each case. If the optical density was 40 or less, positivity was defined as mild; between 40 and 80 was considered moderate, and values of 81 or more were severe. A database was constructed with SPSS, version 20, and descriptive statistics were analyzed by X2 test and comparison of means.

## Results

**Figure 1:**
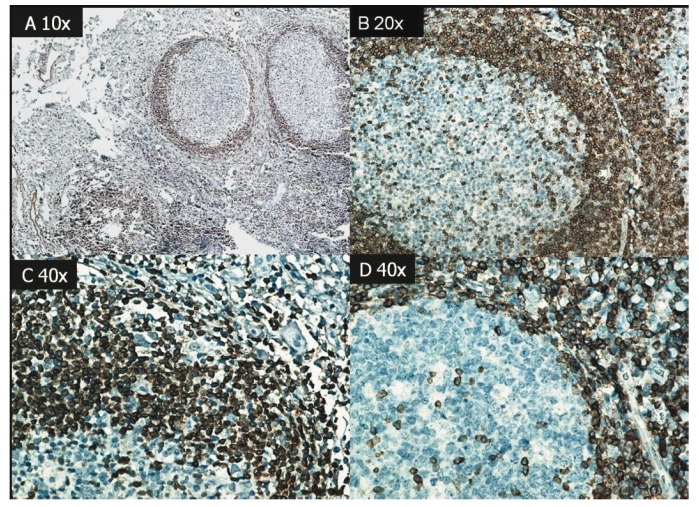
A, B, C, and D. (100x) Cytoplasmic reaction of Bcl-2 antibody in peripheral lymphocytes forming the lymphoid follicle in AP cheilitis and in diffuse lymphocytic infiltrate, indicating an attempt to suppress apoptosis in this site.

A total of 64 cases were processed, of which 40 (62.5%) were cheilitis AP and 24 (37.5%) were AP in the skin. ( Table 1) Of the 40 cheilitis samples, 27 were positive for Bcl-2 and caspase 3 (67.5%), p53 was expressed in 30 (75%), and Bax was present in 1 (2.5%); none was positive for Fas [positivity was limited to internal controls (melanocytes and apoptotic keratinocytes)]. (Fig. 1)

**Table 1:**
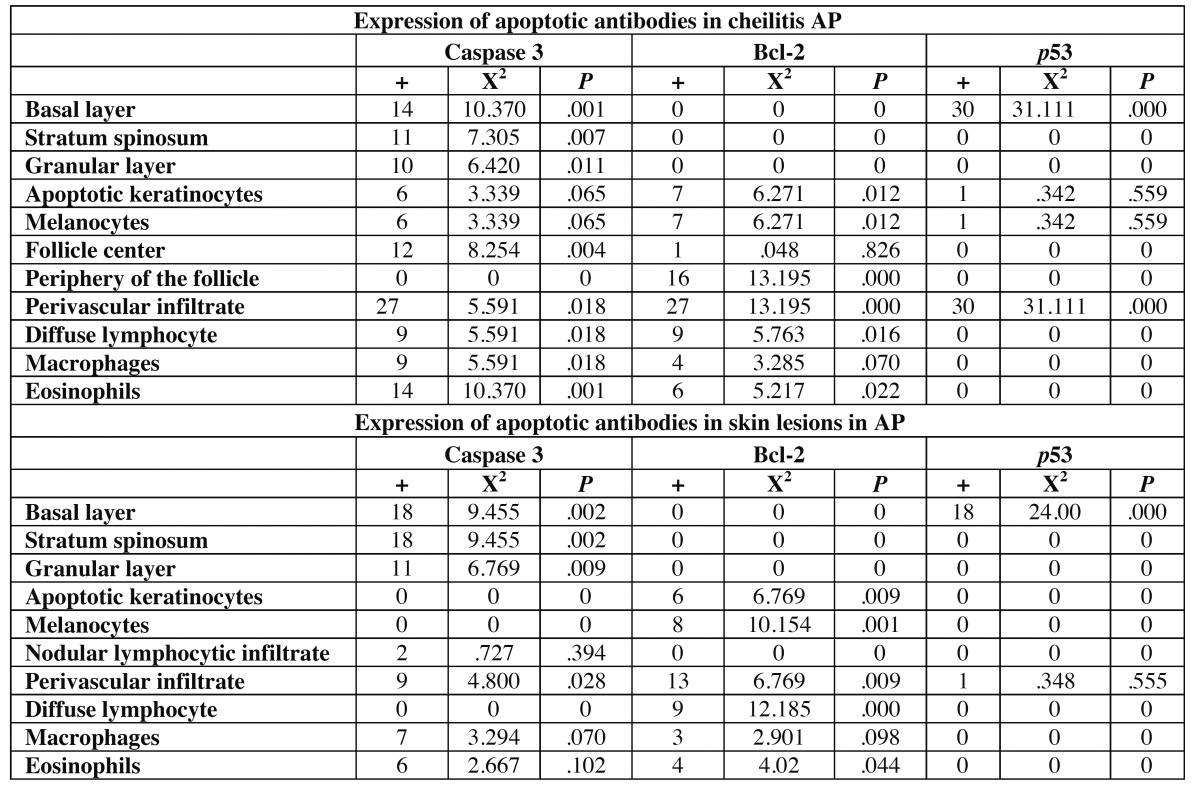
Immunohistochemical analysis in cheilitis AP and skin lesions.

**Figure 2:**
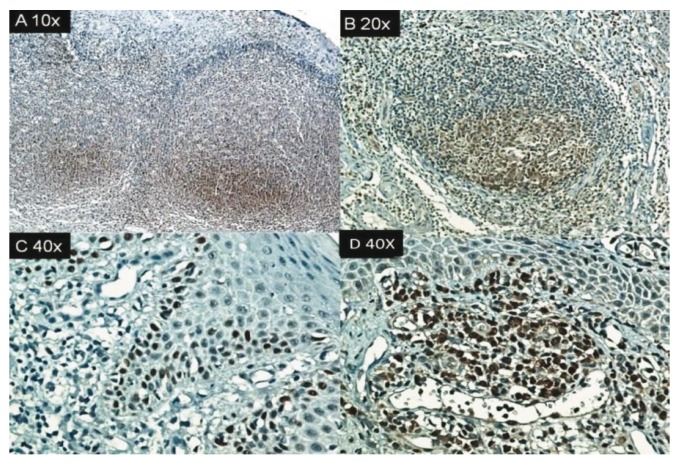
A and B. (100x) Caspase 3, positivity in the central cell of the lymphoid follicle in AP cheilitis. C. (400x) Reaction in the basal layer of skin lesions D. (400x) Perivascular lymphocytic infiltrate. Caspase 3 positivity was nuclear in epithelial cells and cytoplasmic in inflammatory cells; positivity indicates the presence of apoptosis.

**Figure 3:**
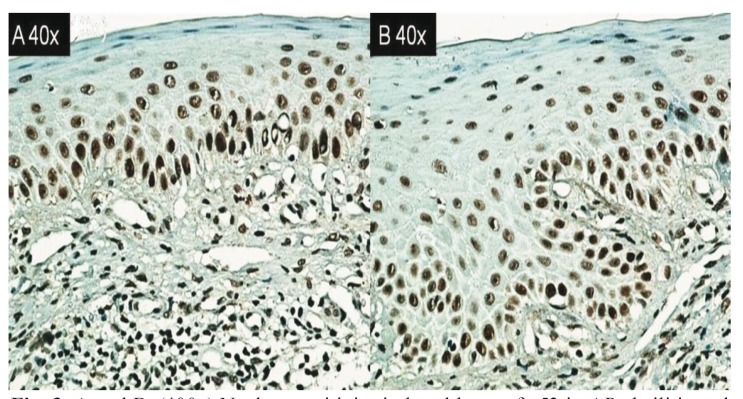
A and B. (400x) Nuclear positivity in basal layer of p53 in AP cheilitis and skin lesions. In cheilitis, the positivity was in the perivascular lymphocytic infiltrate.

Of the skin lesions, p53 and caspase 3 were expressed in 18 of 24 cases (75%), and 13 were positive for Bcl-2 (54%). None was positive for Bax or Fas. (Figs. 2,3).

The mean expression levels for caspase 3, bcl-2, and p53 in the cheilitis samples were 66.61, 116.71, and 38.71, respectively, versus 59.34, 68.54, and 32.71 for skin lesions; no significant differences were noted between these values ( Table 2).

**Table 2:**
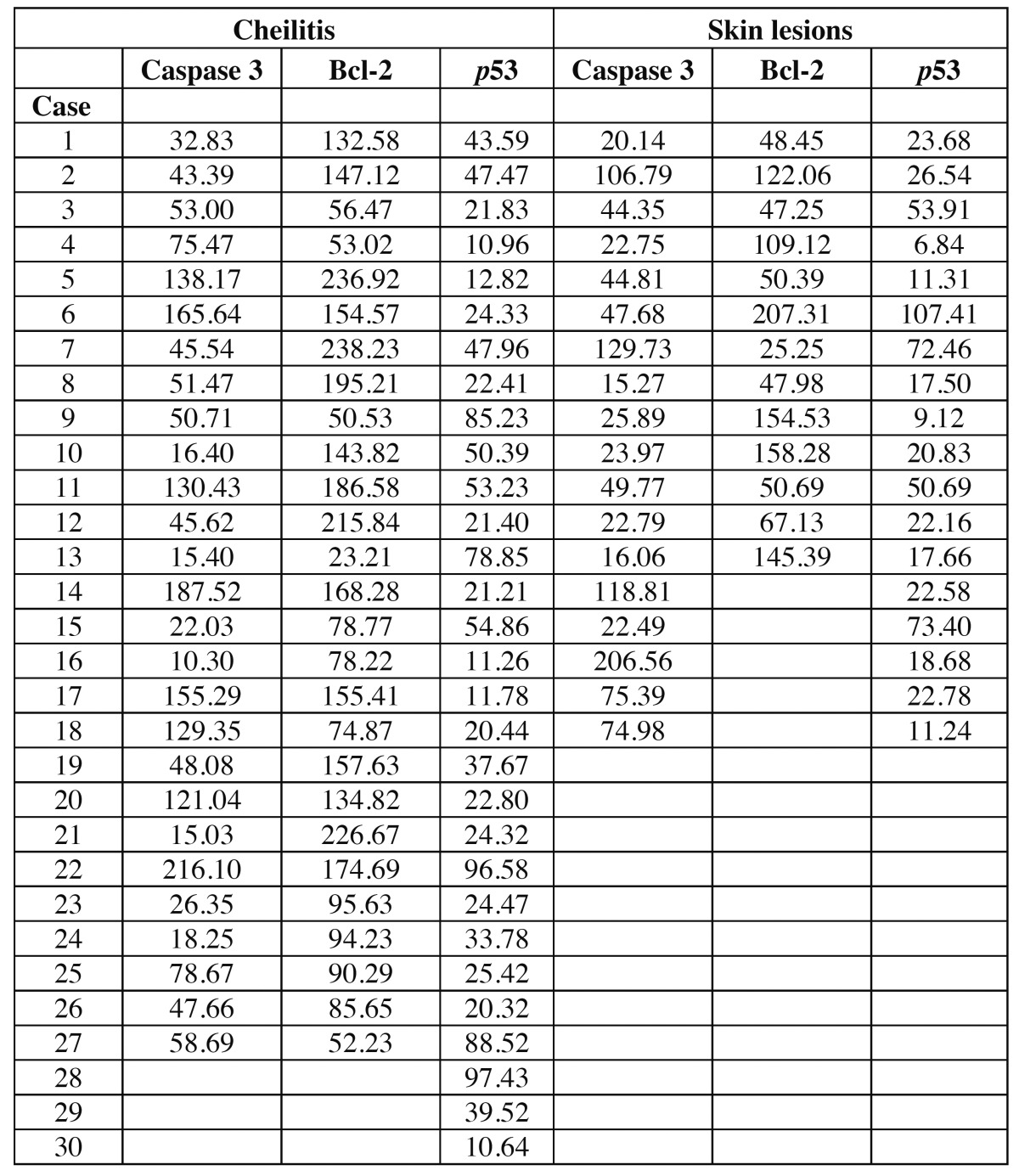
Optical density of apoptotic antibodies in cheilitis and skin while more intensity positive is the case, the software shows a smaller value.

## Discussion

Apoptosis is a physiological death program that can be initiated in 2 ways. The extrinsic pathway, involves a death receptor (TNF type 1) and Fas (CD95) and their ligands, TNF and Fas ligand (Fas L), respectively. In the intrinsic pathway, the cell suffers irreparable damage, and apoptosis is initiated by exposure to antigens, such as bacteria, toxins, and free radicals, and solar radiation and mediated by inhibitors, such as Bcl-2 and bcl-XL, and facilitators, such as Bax and Bad (8,11).

Signaling through Fas leads to apoptosis, requiring the binding of Fas to anti-Fas or cells that expressing Fas L or soluble Fas L. Fas associates with MORT1 (or FADD-Fas-associating protein with death domain) and RIP (receptor-interacting protein). Then, the Fas-FADD complex binds to MACH, activating the latter. MACH is a proteolytic enzyme that belongs to the family of caspases (caspase-8) that trigger apoptosis (12-14).

We observed Fas positivity in apoptotic keratinocytes and melanocytes in cheilitis lesions and skin lesions, although this positivity is considered to be normal (internal control), being induced by the inflammatory infiltrate. There were no Fas-positive cases in characteristic AP histological sites. Thus, we infer that the extrinsic pathway of apoptosis is not active in the pathophysiology of AP.

Bax is a proapoptotic member of the Bcl-2 family, which regulates intrinsic apoptosis signaling (15). It is expressed in the cytoplasm of cells in an inactive form and is stimulated in the early stages of apoptosis, associating with mitochondria through unidentified mechanisms and inducing conformational changes that trigger programmed cell death (16). In this study, we found in one Bax-positive case of cheilitis.

Bcl-2 suppresses apoptosis by 2 mechanisms: direct action against mitochondria to prevent increased permeability and through interactions with other proteins. In other cases, Bcl-2 can also suppress apoptosis by fixing cytosolic proteins and abducting in the mitochondrial membrane (8). In our cheilitis AP samples, we observed significant positivity with anti-Bcl-2 (periphery of lymphoid follicles), reflecting an attempt to suppress apoptosis at this site. Other sites of antibody positivity in both lesion types were the perivascular infiltrate, diffuse lymphocytes, keratinocytes, melanocytes, and macrophages. Bax and Bcl-2 should be interpreted jointly-Bax overexpression in cells accelerates apoptosis in response to a death signal, and in contrast, when Bcl2 is over expressed, cell death is suppressed.

Activation of caspase 3 is a hallmark of apoptosis and can be used to quantify the activation and inhibition of the cell death cascade. Cells become more susceptible to staurosporine (protein kinase inhibitor) in a specific phase of the cell cycle, which leads to cell death (17).

There are 2 major groups of caspases: initiating and executing. Initiator caspases are activated by auto proteolysis when they translocate to specific compartments or through adapters/drivers. Executing caspases are activated by specific cuts that are induced by initiator caspases. These proteases mediate the final cuts in substrates, causing the typical morphology in apoptosis, including signal proteins, DNA repair enzymes, structural proteins, and transcription factors (18), caspase 3 is the executor of apoptosis.

In our tissue samples, caspase 3 expression was nuclear in epithelial cells and cytoplasmic in connective tissue cells. In lymphoid follicles, it was expressed significantly in central lymphocytes, indicating the onset of the cell death cascade. Bcl-2 was positive in peripheral lymphocytes in the follicle, thus, we conclude that apoptosis was initiated in B lymphocytes and inhibited in T lymphocytes.

Caspase 3 was expressed in diffuse and perivascular cells, in the basal layer of the epithelium (where mitosis is frequent), and in the stratum spinosum, indicating that the apoptosis was activated in different histopathological sites.

p53 protein mediates apoptosis in response to DNA-damaging agents. Typically, p53 is a transcription factor that governs the gene expression cluster that arrests the cell cycle or initiates the apoptotic process (19). We noted p53 positivity in AP lesions in the skin and lip.

## Conclusion

Apoptosis was observed in AP, which will help us understand its pathophysiology. We propose that apoptosis is the last step in the type IV subtype a-b hypersensitivity response-activation of the intrinsic pathway indicates that external factors, such as UV-A and -B are the trigger.
